# The comparative effectiveness of two brief tobacco interventions in the U.S. Air Force: Perceived harm and intentions-to-use of tobacco products

**DOI:** 10.18332/tid/87142

**Published:** 2018-06-01

**Authors:** Zoran Bursac, Robert C. Klesges, Melissa A. Little, Brittany D. Linde, Lucy Popova, Cameron M. Kaplan, Gerald W. Talcott

**Affiliations:** 1 Department of Preventive Medicine, University of Tennessee Health Science Center, Memphis, United States; 2 Center for Addiction and Prevention Research, Department of Public Health Sciences, University of Virginia, Charlottesville, United States; 3 Organizational Wellness and Learning Systems, Fort Worth, United States; 4 School of Public Health, Georgia State University, Atlanta, United States

**Keywords:** tobacco, perceived harm, intentions-to-use, comparative effectiveness, effect size

## Abstract

**INTRODUCTION:**

Brief health prevention programs have been shown efficacious in prevention of tobacco use initiation and re-initiation in the US Air Force. In this manuscript we apply a comparative effectiveness assessment of two published studies, based on testing the equality of effect sizes for perceived harm and intentions-to-use for five tobacco products.

**METHODS:**

We calculate and compare the effect sizes from the brief tobacco intervention (BTI) study (N=1055) with those of the anti-tobacco media campaign (MEDIA) study (N=665), for perceived harm and intentions-to-use of cigarettes, smokeless tobacco, cigarillos, e-cigarettes and hookah, among Airmen in the US Air Force Technical Training. Univariate and multivariate parametric and non-parametric methods and models were applied to compare the outcomes between the interventions. In addition, we calculate and report the cost of each intervention per Airman.

**RESULTS:**

Effect sizes for perceived harm were 0.24–0.99 for BTI and 0.17–0.33 for MEDIA, while intentions-to-use effect sizes were 0.14–0.34 for BTI and 0.01– 0.07 for MEDIA, depending on the product. BTI intervention effects sizes were significantly greater than MEDIA intervention for all products, mainly among past users, and for both perceived harm (all p<0.0001) and intentions-to-use (all p<0.01). Cost per Airmen was comparable between the two interventions, $14.90 for BTI and $16.52 for MEDIA.

**CONCLUSIONS:**

Direct comparison suggests that BTI produced effect sizes of significantly higher magnitude in the desired direction for perceived harm and intentions-to-use, for five tobacco products most commonly used by the Airmen, and mainly among past users.

## INTRODUCTION

Tobacco use causes 480 000 deaths each year in the United States^1^. It increases the risk of coronary heart disease and stroke up to 4 times, and lung cancer by a staggering 25 times^1,2^. Young adults (age 18–25 years) are at high risk for tobacco use due to targeting by tobacco companies^3^. In 2013, 37% of 18–25 year olds used some form of tobacco in the past month, compared to 7.8% of 12–17 year olds and 25.7% of 26 years old and older^4^.

Young adults comprise the largest age demographic in the US military^5^. Tobacco prevalence rates are significantly higher among Active Duty military than for the general population^6^. Common tobacco products used in the US Air Force (USAF) are: cigarettes (11.2%), smokeless tobacco (8.6%), cigarillos (8.7%), hookah (10.4%) and e-cigarettes (6%)^7^.

Military training and service begins with 10.5 weeks of forced abstinence. At the end of this period, nearly 2/3 of trainees are confident that they won’t return to tobacco products, although the majority do^7^. In fact, nearly 70% of tobacco initiation/re-initiation occurs during Technical Training^8^. This presents an opportunity for a teachable moment that should not be missed.

There are different brief health prevention programs that may be effective for new recruits in the USAF, addressing this challenge and aiming at preventing tobacco use initiation and/or reinitiation^9^. One type is face-to-face behavioral intervention that is more time intensive and interactive. Another is anti-tobacco media messages, which require little time and resources. Research evaluating the effectiveness of anti-tobacco media campaigns on young adults, and in particular on the military population, is rare^10-12^.

Our research group has tested two such approaches in the USAF, with a goal of reducing tobacco use following the tobacco ban; a brief tobacco intervention (BTI) and anti-tobacco media campaign messages (MEDIA). Both studies measured intervention impact on short-term proxy outcomes of perceived harm from tobacco products, and intentions-to-use tobacco products in the next 12 months. Little et al. tested the BTI (N=1055) among the USAF trainees and reported that it significantly increased perceived harm and decreased intentions-to-use any of the nine tobacco and nicotine containing products examined in their study^13^. In the MEDIA study (N=782), Popova et al. concluded that anti-smoking advertisements produced for the general public might also be effective with a young adult military population and have additional effects on perceptions of harm and intentions-to-use of other tobacco and nicotine containing products other than cigarettes^14^.

The BTI intervened with a brief tobacco use prevention program based on motivational interviewing^13^, and MEDIA with a combination of existing anti-tobacco media messages^14^. Given the similarity between the two projects, and the fact that both interventions are brief, conducted on the same population of Air Force trainees, the same location during approximately the same time, measuring the same outcomes and the same pre-post design, we opportunistically conduct a secondary comparative effectiveness analysis to test the effect sizes of the interventions head-to-head. Even though both studies found changes in perceived harm and intentions-to-use, we aim to determine which, if either, had a higher effect in terms of short-term proxies of five tobacco products, most commonly used among the Airmen. This will allow us to make recommendations on which intervention to use on USAF trainees, given limited resources. Also, it will provide primary data for future comparative studies targeting tobacco abstinence, such as trials comparing behavioral intervention, media intervention or a combination of the two.

## METHODS

In the following sections we provide a short overview of participants, design, measures and interventions for the BTI and MEDIA studies, along with a discussion of similarities and differences between the two. Additional details are provided by Little et al.^13^ and Popova et al.^14^.

### Subjects

BTI participants were 1055 USAF Airmen in Technical Training at the Joint Base San Antonio–Lackland Air Force Base in San Antonio, Texas, from October 2014 to March 2015. Similarly, MEDIA participants were 782 Airmen in Technical Training at the Joint Base San Antonio–Lackland Air Force Base/Ft. Sam Houston, September and October 2014. For the purposes of the present work, we only included 665 participants from the MEDIA study that were randomized to active intervention. Participation consent rates were 99% and 98%, for BTI and MEDIA studies, respectively. The protocols for both studies were approved by the Institutional Review Board at Wilford Hall Ambulatory Surgical Center, but were adjudicated exempt because all responses were anonymous.

### Design and measures

The BTI study was designed as a one-arm comparison with no control group, measuring mentioned proxy outcomes pre- and post-intervention. The MEDIA study had 5 arms (media themes) and the control group, similarly measuring proxy outcomes pre- and post-intervention. Both projects share the similarity that main outcome measures were changes from pre-test to post-test in perceived harm and intentions-to-use of common tobacco products. These outcomes have been shown to be good predictors or proxies for future behavior in general^15-22^. The BTI study targeted 4 tobacco products (cigarettes, smokeless tobacco, hookah and e-cigarettes) and measured outcomes on 9, while MEDIA messages targeted cigarettes and second hand smoke, while measuring the outcomes on 5 tobacco products. Therefore, here we compare the effect sizes for the 5 products that were common to both studies: cigarettes, smokeless tobacco, cigarillos, e-cigarettes and hookah. These products are also most commonly used among the Airmen^7^. In both studies Airmen were asked to rate the harm of each of the products, and to indicate the likelihood of use within the next 12 months. In the BTI study, both scales ranged from 1–7. In the MEDIA study, the harm scale ranged from 1–9 and the intent scale from 0–100 in 10-point increments. Below we describe the linear transformation that was applied to make the scales comparable.

### Interventions

The BTI intervention is based on prior research and tobacco control programs in the military^7,23-26^. It is approximately 40 minutes long, delivered in a group setting, interactive with the participants and based on principles of motivational interviewing^13,27^. The MEDIA intervention used existing print and video anti-tobacco advertisements, developed by the California Department of Public Health and Rescue Social Change Group, which have undergone qualitative and quantitative testing^28,29^. Five advertisement themes consisted of anti-industry, health effects + anti-industry, sexual health effects, second hand smoke, and environment + anti-industry messages. The length of the study session was approximately 45 minutes long (including pre-test, post-test and exposure to 4 ads, each one minute long) and delivered in the group setting, but not interactive with the participants^14^. For the purposes of this comparison, we collapsed the results of the MEDIA study across all five themes, but present and discuss the effects of individual themes on the measured outcomes.

### Statistical methods and analysis

#### Linear transformation of scales

Likert scales in the BTI study measuring perceived harm and intentions-to-use ranged from 1–7. In the MEDIA study Likert scale for perceived harm ranged from 1–9, and for intentions-to-use from 0–100 in 10-point increments. In order to make the scales comparable we performed a linear transformation of two scales from the MEDIA study so that all scales ranged from 1–7. To accomplish that we used the following linear transformation:

*Y = (B-A)*x*(X-a)/(b-a) + A,*

where *Y* is a desired vector with new minimum *A* and maximum *B*, and *X* is the original vector with minimum *a* and maximum *b*.

For example, in converting MEDIA harm scale that ranged from 1–9 to comparable BTI scale with range from 1–7, the calculations were as follows:

*Y = (7-1)*x*(X-1)/(9-1) + 1 = 6(X-1)/8 + 1*, hence if *X=1* then *Y=1*, and if *X=9* then *Y=7*.

#### Cohen’s effect size d

In many common experimental designs the power of a test is determined by four factors: Level of significance *α*, sample size *n*, population standard deviation *σ*, and difference between the means Δ.

When trying to determine the appropriate sample size for a randomized study, researchers need to know or assume the above parameters to estimate the sample size; however, often these are not known either based on previous research or available literature. In situations when this is the case, we can divide the hypothesized absolute value of the difference in means by the population standard deviation, resulting in a standardized positive score called effect size^30^, denoted as *d*:

*d =* |*µ-µ_0_*|/*σ*,

where a mean is represented by *µ*. It expresses the magnitude of the difference in means in standard deviation units. Based on Cohen, a *d* of 0.2 is considered a small effect, 0.5 a medium effect, and ≥0.8 a large effect^30^. The described approach was used to calculate the effect sizes from the two brief tobacco interventions, relative to perceived harm and intentions-to-use for five most common tobacco products, and can be used in future research and to determine the relative short-term efficacy of the two separate approaches.

### Data analysis

All analyses were performed with SAS/STATv14.1 (SAS Institute Inc., Cary, NC). Descriptive statistics including means, standard deviations and proportions were generated for comparable demographic and socioeconomic characteristics for both samples. Equality of the mentioned proportions were tested with a chi-squared test. Starting with the methods described above (linear transformations of the scales), we calculated the change in perceived harm and intentions-to-use for the five products of interest, along with the standard deviation, and finally a resulting effect size d. It is important to point out that although the effect size d is an absolute value of the change, and therefore it is always positive, a decrease in intentions-to-use a product, as a result of the intervention, is a negative number. The true directionality for intentions-to-use is presented as *Δ* in Tables 3 and 7, but as *β* in Table 4. Significance of the effect size within each study and within each MEDIA theme was tested with a non-parametric Wilcoxon Signed Rank test for paired observations. Univariate comparison of effect sizes between two studies was performed with non-parametric Wilcoxon Man-Whitney U two sample test. Moreover, for comparison of BTI effects to individual MEDIA themes, we applied a Kruskal-Wallis test for six independent samples, with the Dwass Steel Critchlow-Flinger (DSCF) adjustment method for multiple comparisons^31-33^. Finally, we fit a multivariable linear regression model for all outcome measures individually, to estimate the study effect while adjusting for comparable demographic covariates, which include gender, race and Hispanic ethnicity. Furthermore, we tested interactions between the study and demographic variables, as well as ever use of any tobacco product. All of the differences and associations were considered significant at the alpha level of 0.05.

### Cost analysis

We estimated the cost per Airmen for each of the interventions based on the number of Airmen, length of participation, and the amount of time interventionists and clinical psychologist put into training, delivery and supervision.

## RESULTS

### Demographics

The BTI sample (N=1055) had 77% men compared to 69% in MEDIA (p=0.0002). Samples did not differ with respect to race distribution or Hispanic ethnic descent. In the BTI sample, average age was 20 years (SD=2.5), 9% were married and 64% completed high school or GED. The MEDIA study (N=655) did not collect these three demographic measures, but the sample came from the same population of USAF trainees at the same training base within a few months of each other (Table 1).

**Table 1:** Comparison of demographic characteristics between two studies

	*BTI (N=1055)*	*MEDIA (N=665 )*	*p*
**Age**	20.1 (2.5)	Na	na
**Married**	9.3	Na	na
**HS/GED**	63.6	Na	na
**Male**	77.4	69.1	0.0002
**Race**			0.519
**W**	68.1	68.2	
**B**	14.4	16	
**O**	17.5	15.8	
**Hispanic**	18	17.8	0.9348

na – comparison is not possible since MEDIA study did not collect these variables

### Perceived harm

Table 2 summarizes the effect sizes of perceived harm for each product by study, and the comparison of the effects between the studies.

**Table 2:** Comparison of effect sizes for perceived harm of tobacco products between two studies

*Harm*	*BTI (N=1055 )*			*MEDIA (N=665 )*			*p*
*Overall*	*Δ*	*SD_Δ_*	*d*	*Δ*	*SD_Δ_*	*d*	
Cigarettes	0.222	0.921	0.241	0.092	0.547	0.168	<0.0001
ST	0.593	1.202	0.494	0.160	0.793	0.202	<0.0001
Cigarillos	0.605	1.353	0.447	0.152	0.731	0.208	<0.0001
E-cig	1.513	1.843	0.821	0.331	1.013	0.327	<0.0001
Hookah	2.109	2.111	0.999	0.261	0.956	0.273	<0.0001

Δ – change in perceived harm from pre- to post-test. SDΔ – standard deviation of the change. d – effect size. E-cig: e-cigarette, ST: smokeless tobacco.

In the BTI study, effect sizes for perceived harm ranged from 0.24 for cigarettes to 0.99 for hookah. Effects for smokeless tobacco, cigarillos and e-cigarettes were in between, at 0.49, 0.45 and 0.82, respectively. All were statistically significant (all p<0.0001) (Table 2).

In the MEDIA study effect sizes ranged from 0.17 for cigarettes to 0.33 for e-cigarettes. Smokeless tobacco, cigarillos and hookah effects were 0.2, 0.21 and 0.27, respectively. These were all statistically significant effects as well (all p<0.0001) (Table 2). Individual MEDIA themes also had a significant effect on most of the tobacco products with some, such as sexual health, as high as 0.47 and 0.53 for e-cigarettes and hookah, respectively (see Appendix Table 6 in Supplementary file).

Univariate comparison of effect sizes between two samples showed that BTI had significantly higher effects for perceived harm than MEDIA, for all tobacco products (all p<0.0001) (Table 2). Similar findings were observed for comparisons between BTI and individual MEDIA themes (see Appendix Table 6 in Supplementary file).

### Intentions-to-use in next 12 months

Table 3 summarizes the effect sizes of intentions-to-use in the next 12 months for each product by study, and the comparison of the effects between the studies.

**Table 3:** Comparison of effect sizes for intentions-to-use tobacco products between two studies

*Intent*	*BTI (N=1055 )*			*MEDIA (N=665 )*			*p*
*Overall*	*Δ*	*SD_Δ_*	*d*	*Δ*	*SD_Δ_*	*d*	
**Cigarettes**	-0.118	0.860	0.138	-0.020	0.416	0.048	0.0025
**ST**	-0.180	0.881	0.204	0.008	0.372	0.022	<0.0001
**Cigarillos**	-0.208	1.021	0.204	-0.006	0.695	0.008	0.0001
**E-cig**	-0.116	1.100	0.106	0.048	0.739	0.065	0.0006
**Hookah**	-0.489	1.437	0.341	-0.014	0.714	0.019	<0.0001

Δ – change in perceived harm from pre- to post-test. SDΔ – standard deviation of the change. *d* – effect size. E-cig: e-cigarette, ST: smokeless tobacco.

In the BTI study effect sizes for intentions-to-use ranged from 0.14 for cigarettes to 0.34 for hookah. Effects for smokeless tobacco, cigarillos and e-cigarettes were in between, at 0.2, 0.2 and 0.11, respectively. All were statistically significant (all p<0.0001) (Table 3).

In the MEDIA study effect sizes ranged from 0.01 for cigarillos to 0.07 for e-cigarettes. Cigarettes, smokeless tobacco and hookah effects were 0.05, 0.02 and 0.02, respectively. These effects were all non-significant (Table 3). Even though the effects for intention collapsed across all five advertisement themes in the MEDIA study and were not statistically significant, several individual MEDIA themes such as health effects, sexual health and environmental impact, did produce significant effects for certain products, as presented in the original empirical paper^14^ (see Appendix Table 7 in Supplementary file).

Similar to perceived harm, univariate comparison of effect sizes between two samples showed that BTI had significantly higher effects on intentions-to-use than MEDIA, for all tobacco and nicotine containing products (all p<0.01) (Table 3). Similar findings were observed for comparisons between BTI and several individual MEDIA themes and tobacco products (see Appendix Table 7 in Supplementary file).

### Multivariable regression

Table 4 shows the results from the adjusted regression models for both harm and intentions-to-use, for all five products. The findings support the univariate comparisons presented in Tables 2 and 3. BTI had a significantly larger increase in effects for perceived harm and decrease for intentions-to-use compared to MEDIA, for all products except cigarettes, after the adjustments for demographic covariates. Whites were associated with significantly higher effects of perceived harm for cigarillos, while Blacks and Hispanics were associated with significantly higher effects of intentions-to-use cigarettes and smokeless tobacco, respectively. There are several other borderline significant associations (p<0.1) that was much larger among past users. are presented in Table 4, but not discussed here.

**Table 4:** Regression models estimating the BTI vs MEDIA effect, adjusting for demographics

*Harm*	*Cigarettes d*			*ST d*			*Cigarillos d*			*E-cigarettes d*			*Hookah d*		
	*β*	*SE*	*p*	*β*	*SE*	*p*	*β*	*SE*	*p*	*β*	*SE*	*p*	*β*	*SE*	*p*
**BTI**	0.088	0.053	0.0936	0.292	0.053	<0.0001	0.249	0.053	<0.0001	0.4973	0.055	<0.0001	0.7692	0.055	<0.0001
**Male**	-0.003	0.059	0.9572	0.114	0.059	0.0534	0.015	0.059	0.7945	-0.041	0.061	0.4971	-0.035	0.062	0.5661
**Hispanic**	-0.065	0.073	0.3736	-0.134	0.074	0.0669	0.071	0.074	0.3414	0.016	0.077	0.8349	0.112	0.078	0.1477
**Race**															
**W**	0.001	0.073	0.9851	-0.055	0.073	0.4529	0.176	0.074	0.0171	0.036	0.077	0.6432	0.033	0.078	0.6667
**B**	0.107	0.095	0.2603	-0.069	0.095	0.4705	0.154	0.095	0.1072	0.077	0.099	0.4354	0.158	0.101	0.1184

**Intent**															

**BTI**	-0.073	0.052	0.1558	-0.206	0.051	<0.0001	-0.179	0.052	0.0006	-0.158	0.051	0.0021	-0.315	0.053	<0.0001
**Male**	0.006	0.058	0.9227	-0.111	0.057	0.0518	-0.103	0.058	0.0779	-0.056	0.058	0.3119	0.04	0.059	0.4957
**Hispanic**	0.039	0.071	0.9227	0.1613	0.071	0.0228	0.086	0.072	0.2328	0.024	0.071	0.7396	-0.069	0.073	0.3433
**Race**															
**W**	0.078	0.072	0.2752	-0.049	0.071	0.4917	-0.047	0.072	0.514	0.052	0.072	0.4703	0.096	0.073	0.189
**B**	0.195	0.093	0.036	0.156	0.092	0.0914	-0.047	0.094	0.613	0.017	0.093	0.8513	0.097	0.095	0.3112

*β* for BTI indicates larger increase in effect size for perceived harm and decrease in intentions-to-use after the adjustment.

While there were no significant interactions between the study and demographic variables, we did detect significant interactions between the study and ever use. The results are shown in Table 5. BTI had significantly higher effects for perceived harm and decrease for intentions-to-use compared to MEDIA, among those who were past users, for all products including the cigarettes, after the adjustments for demographic covariates. BTI also had significant effects for e-cigarette perceived harm and hookah intention never users, but the magnitude of the effect was much larger among past users.

**Table 5:** Regression models estimating the BTI vs MEDIA effect for never and ever users, adjusting for demographics

*Harm*	*Cigarettes d*			*ST d*			*Cigarillos d*			*E-cigarettes d*			*Hookah d*		
	*β*	*SE*	*P*	*β*	*SE*	*p*	*β*	*SE*	*p*	*β*	*SE*	*p*	*β*	*SE*	*p*
**Never users**	-0.048	0.089	0.5839	0.077	0.084	0.3599	0.057	0.088	0.516	0.296	0.09	0.001	0.487	0.093	<0.0001
**Ever users**	0.179	0.066	0.007	0.429	0.069	<0.0001	0.382	0.068	<0.0001	0.625	0.071	<0.0001	0.971	0.069	<0.0001

**Intent**															

**Never users**	-0.08	0.037	0.8261	-0.002	0.037	0.9507	0.009	0.038	0.8014	-0.027	0.044	0.5364	-0.089	0.044	0.0456
**Ever users**	-0.166	0.084	0.049	-0.391	0.083	<0.0001	-0.384	0.084	<0.0001	-0.265	0.082	0.0013	-0.566	0.083	<0.0001

### Cost

Airmen time was valued using Department of Defense (DoD) Military Personnel Composite Standard Pay and Reimbursement Rates for E-1 ($20/hour). Interventionist ($28.95/hour) and clinical psychologist ($72/hour) time was valued using UTHSC payroll records. We estimated the time of Airmen participation based on the number of Airmen that participated and the duration of intervention in hours. Average cost of the BTI per Airman was $14.90, and for MEDIA $16.52 (see Appendix Table 8 in Supplementary file).

## DISCUSSION

BTI intervention resulted in significant effects for perceived harm, which ranged from small to large effect sizes. Effect sizes for smokeless tobacco and cigarillos were in the medium range of 0.5, but for e-cigarettes and hookah effects were greater than 0.8 indicating large effect size. MEDIA intervention also resulted in significant effects for perceived harm, which were mainly in the small range, with e-cigarettes and hookah experiencing largest impact at 0.33 and 0.27, respectively. In a head-to-head comparison BTI intervention effects sizes were significantly greater than MEDIA intervention.

Regarding intentions-to-use products in the next 12 months, the BTI intervention had mainly small to moderate significant effects. Largest impact was on lowered intentions-to-use hookah at 0.34. Overall the MEDIA intervention had very small non-significant effects on intentions-to-use of any product examined in this comparison, though several advertisement themes within the MEDIA study did produce significant effects, which supports published findings^14^. Similar to perceived harm, BTI intervention effects sizes were significantly greater than the MEDIA intervention (overall or individual themes).

It is important to note that BTI intervention produced significantly larger changes compared to MEDIA, mainly among those who reported past use including cigarettes. It also had significantly larger effects for e-cigarette perceived harm and hookah intent never users.

Evidence suggests that BTI produced larger effects across the board, overall and for past users, for both perceived harm and intentions-to-use. The BTI intervention was interactive and more intensive while that of MEDIA was not interactive and consisted of short-time exposure to ads. Some studies report that personal interactive interventions are more effective with respect to behavioral outcomes. The BTI intervention used the Socratic teaching style and garnered participation through the principles of motivational interviewing, which has been associated with improved treatment effects in general and for tobacco outcomes specifically^27,34,35^. It is also important to note that BTI intervention targeted four products compared in this study, while perhaps MEDIA intervention could have been diluted given the evaluation of various message themes and sole target on cigarettes. More focused and time intensive MEDIA interventions that use only the highest rated ads along with systematic and targeted approach on multiple tobacco products could potentially result in higher effects and this should be explored in future studies. Initial perceived harm for cigarettes was high, and intentions-to-use in the future low (ceiling and floor effects), therefore it is somewhat difficult to achieve an increase or decrease as a result of intervention. The largest intervention effects are observed for hookah, and e-cigarettes to some extent. These products are emerging in popularity and social use and tend to be perceived as less or even harmless^7,23^. BTI intervention had a noticeably large effect on increasing a perception of harm for these two products among past users and nonusers.

Cost of each intervention was approximately $15 per Airman and did not differ significantly between BTI and MEDIA. This suggests that perhaps BTI is more cost effective.

### Limitations

It is important to disclose several limitations of this study, given it was opportunistic and not planed apriori, and how we attempted to minimize their effect on the reliability of the findings. This study was not a randomized clinical trial, rather a comparison of two very similar but not identical military populations, coming from the same military base and approximately during the same period. Not all demographic variables were collected in both samples. MEDIA did not collect age, education and marital status. Variables that were comparable to both studies were gender, race and ethnicity, therefore these are included in the multivariable adjustments. The summary statistics for the other three are presented for the BTI study in Table 1, in order to give the readers an idea about these population characteristics, which are very similar to our other published findings and representative of Airmen in Technical Training^7,23-26^. The timing of the execution of these projects was not identical but it did overlap, with MEDIA being conducted from September-October 2014, and BTI from October 2014-March 2015. While the temporality could have some effect on the outcomes, this is likely to be very small if any. Though all participants in the MEDIA intervention were exposed to four ads within each theme, themes did differ as described above^14^. Different themes had differential impact on the outcomes, yet MEDIA effect sizes are based on the average effects across the different themes. However, even when themes were evaluated individually, the finding that BTI had significantly larger effects was consistent with the results of the analysis when the themes were combined. Effect sizes for the individual themes are presented in the Appendix, especially to show that several themes resulted in small but significant effects regarding intentions-to-use certain products. Ads used as part of the MEDIA intervention mainly targeted cigarettes and some but not all involved military themes^14^. Harm and intent questions were the same but the scales between the studies were different. We did apply linear transformations, as described in the methods section, to make the scales comparable with the same range. We used the outcome measures for the tobacco products that were the same for both studies. Outcome measures of perceived harm and intentions-to-use are proxy measures of hard outcomes such as abstinence. Research has found that intentions are reliable predictors of future use^36^. A number of studies have found that intentions-to-use cigarettes predict initiation or escalation of use among adolescents^17,19-21^. Disproportionate perceptions of harm regarding tobacco products have been observed between smokers and non-smokers^18^. Taken together, these two measures seem to be important predictors of future use. Future studies should evaluate the cost of these interventions in more detail and express it in terms of quality-adjusted life years saved especially for outcomes, such as abstinence, that we did not collect in these two pilot projects.

### Strengths

Despite the limitations, this study inherently possesses a number of strengths that we feel outweigh the weaknesses. As mentioned earlier, this paper presents results from a secondary opportunistic comparison of two large samples of Airmen with a total of 1720 participants, which allows for sufficient power to detect even small differences in effects. Both of these studies are the first of their kind in evaluations of interventions designed to impact multiple tobacco products in a military population. Both interventions significantly improved the outcomes under investigation for those products that were targeted, as well as several others. Direct comparison of these has never been examined, therefore it provides practitioners in this field not just with information and data on individual intervention performance but also with insights on where improvements can be made and the next steps in the research process designed to better the health and readiness of Airmen.

Both studies are very similar in a large population of Airmen in the same location where they were recruited. Methods for these studies include baseline assessment followed by the short tobacco intervention and post assessment. While BTI study collected information on perceived harm and intentions-to-use of 9 tobacco and nicotine containing products, 5 most commonly used products overlapped for both studies, allowing us to compare the effect sizes for those as the outcome measures. Finally, the results of the analysis allow us to make recommendations to researchers and USAF on which intervention to use conditional on resources, and to suggest future work in comparing these interventions for tobacco abstinence.

## CONCLUSIONS

While overall both of these studies resulted in significant effects, direct comparison suggests that BTI produced the effect sizes of significantly higher magnitude in the desired direction for perceived harm and intentions-to-use, for five tobacco products most commonly used by the Airmen, and mainly among past users. In addition, it happened at a comparable cost. Future research should examine the effect that a more focused and time-intensive MEDIA intervention, consisting of only the most effective ads targeting all products, has on the outcomes of interest presented here. We should also evaluate the effects of both of these interventions on concrete behavioral outcomes such as tobacco abstinence. This potentially sets up a comparative effectiveness trial in the military that can test behavioral intervention versus the media intervention, or versus the combination of both. Given similar cost between interventions, USAF should use the behavioral intervention to prevent tobacco initiation and re-initiation after a mandatory ban; however, even a brief media-based intervention, such as showing trainees anti-smoking tobacco ads for a short period of time, might have positive effects.
