# Supplementary material for: The comparative effectiveness of two brief tobacco interventions in the U.S. Air Force: Perceived harm and intentions-to-use of tobacco products
Source: Tob Induc Dis. 2018 Jun 1;16:26. doi: 10.18332/tid/87142 (PMC6659509; doi:10.18332/tid/87142)
Supplement: Supplementary file 1 [file TID-16-26-s1.pdf]

APPENDIX

Table 6. Significance of each product perceived harm effect size within the theme and comparison to BTI

MEDIA theme - Harm	Product	*d*	p*	p**
Anti-industry	Cigarettes	0.159	0.0153	0.0639
	ST	0.062	0.1273	<0.0001
	Cigarillos	0.062	0.4647	<0.0001
	E-cig	0.326	<0.0001	<0.0001
	Hookah	0.125	0.055	<0.0001
Health effects	Cigarettes	0.328	0.0005	0.6043
	ST	0.375	0.0008	0.0017
	Cigarillos	0.241	0.025	0.013
	E-cig	0.354	0.0003	<0.0001
	Hookah	0.278	0.0003	<0.0001
Sexual health	Cigarettes	0.025	0.672	0.0018
	ST	0.098	0.158	0.0006
	Cigarillos	0.329	0.0002	0.3928
	E-cig	0.469	<0.0001	<0.0001
	Hookah	0.534	<0.0001	<0.0001
Secondhand smoke	Cigarettes	0.147	0.1684	0.2697
	ST	0.174	0.0387	0.0937
	Cigarillos	0.239	0.0377	0.1663
	E-cig	0.262	0.0026	<0.0001
	Hookah	0.308	0.0118	<0.0001
Environmental impact	Cigarettes	0.193	0.0133	0.5177
	ST	0.337	0.0002	0.1214
	Cigarillos	0.263	0.0016	0.0121
	E-cig	0.215	0.0148	<0.0001
	Hookah	0.254	0.0061	<0.0001
BTI	Cigarettes	0.241		
	ST	0.494		
	Cigarillos	0.447		
	E-cig	0.821		
	Hookah	0.999		

d - effect size each theme had on the perceived harm of the individual tobacco product.

*Wilcoxon Signed Rank test - testing the significance of each product effect size within the theme.

**Kruskal-Wallis test comparing BTI to individual MEDIA themes with DSCF adjustments for multiple comparisons; significant p-values indicate larger effect size for perceived harm in the BTI study for a given product.

Table 7. Significance of each product intentions-to-use effect size within the theme and comparison to BTI

MEDIA theme - Intention	Product	*(Δ)d*	p*	p**
Anti-industry	Cigarettes	0.052	0.3823	0.0683
	ST	0.058	0.5684	0.0053
	Cigarillos	0.04	0.4775	0.0017
	E-cig	0.087	0.1051	0.021
	Hookah	0.056	0.5763	<0.0001
Health effects	Cigarettes	(-)0.146	0.1445	0.8608
	ST	(-)0.119	0.0469	0.7211
	Cigarillos	0.122	0.188	0.0134
	E-cig	0	0.7268	0.4029
	Hookah	(-)0.029	0.4467	0.0078
Sexual health	Cigarettes	(-)0.106	0.0469	0.9997
	ST	(-)0.044	0.5313	0.4488
	Cigarillos	(-)0.208	0.0011	0.9999
	E-cig	0.037	0.6669	0.9818
	Hookah	(-)0.339	0.0001	0.9988
Secondhand smoke	Cigarettes	(-)0.109	0.5785	0.6213
	ST	(-)0.053	0.3125	0.4411
	Cigarillos	(-)0.15	0.2362	0.5103
	E-cig	0.131	0.3597	0.7755
	Hookah	0.119	0.0483	<0.0001
Environmental impact	Cigarettes	0	0.6743	0.4429
	ST	0.236	0.0469	0.0018
	Cigarillos	0.057	0.0847	0.117
	E-cig	0.144	0.3333	0.3881
	Hookah	0.048	0.8135	0.0004
BTI	Cigarettes	(-)0.138		
	ST	(-)0.204		
	Cigarillos	(-)0.204		
	E-cig	(-)0.106		
	Hookah	(-)0.341		

Δ - indicates the direction of change in intentions-to-use; negative sign reflects decrease in intentions.

d - effect size each theme had on the intention to use individual tobacco product.

* Wilcoxon Signed Rank test - testing the significance of each product effect size within the theme.

**Kruskal-Wallis test comparing BTI to individual MEDIA themes with DSCF adjustments for multiple comparisons; significant p-values indicate larger decrease in the effect size for intentions to use in the BTI study for a given product.

Table 8. Estimated costs of the BTI and MEDIA interventions per Airman

	BTI	MEDIA
Number of Airmen	1055	665
Group-Based Brief Tobacco Intervention		
Interventionist hours		
Training	8	2
Intervention delivery	33	27
Clinical Psychologist hours		
Training	1.5	0
Supervision	4	0
Airmen hours		
Intervention participation	1055	665
Total Airmen hours	707	499
Total Hours	753.5	528
Total Cost of Group Sessions[a]	$15723	$10820
Total Cost per Airman	$14.90	$16.52

[a] Airmen time valued using Department of Defense (DoD) Military Personnel Composite Standard Pay and Reimbursement Rates for E-1 ($20/hour); Interventionist ($28.95/hour) and clinical psychologist ($72/hour) time valued using UTHSC payroll records.
